# Supplementary figures and images for: The Young Carers’ Journey: A Systematic Review and Meta Ethnography
Source: Int J Environ Res Public Health. 2022 May 10;19(10):5826. doi: 10.3390/ijerph19105826 (PMC9140828; doi:10.3390/ijerph19105826)

**Figure S1.** PRISMA Flow Diagram.

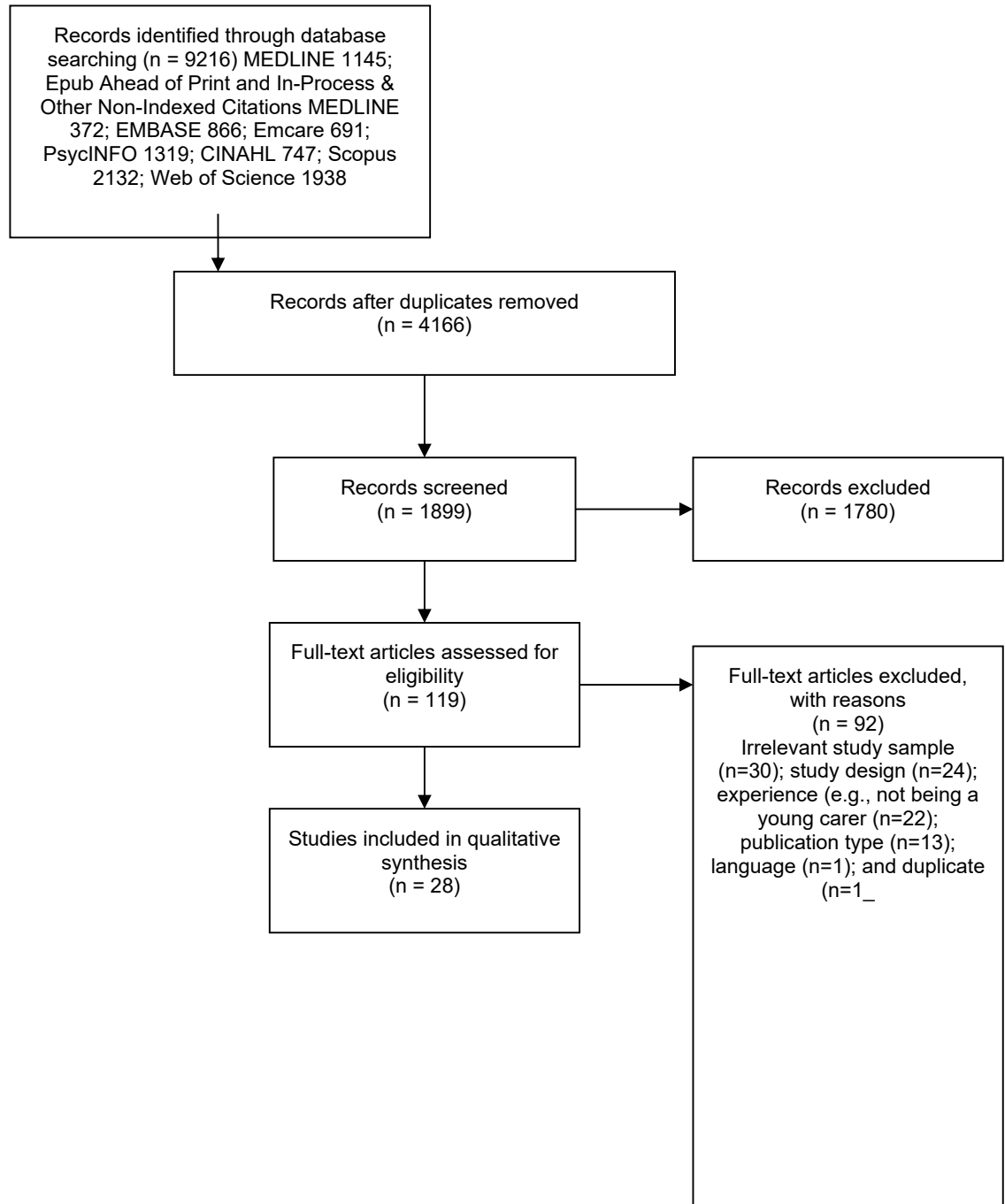

Supplement: Supplementary file 1 [file ijerph-19-05826-s001.zip › ijerph-1645883-supplementary.pdf]
